# Four-Year Incidence of Diabetic Retinopathy in a Spanish Cohort: The MADIABETES Study

**DOI:** 10.1371/journal.pone.0076417

**Published:** 2013-10-17

**Authors:** Miguel Á. Salinero-Fort, Francisco Javier San Andrés-Rebollo, Carmen de Burgos-Lunar, Francisco Jesús Arrieta-Blanco, Paloma Gómez-Campelo, on behalf of MADIABETES Group

**Affiliations:** 1 Fundación de Investigación Biomédica, Hospital Carlos III, Servicio Madrileño de Salud, Madrid, Spain; 2 Centro de Salud Las Calesas, Servicio Madrileño de Salud, Madrid, Spain; 3 Unidad de Epidemiología Clínica, Hospital Carlos III, Servicio Madrileño de Salud, Madrid, Spain; 4 Servicio de Endocrinología y Nutrición, Unidad de Nutrición, Obesidad y Metabolismo, CIBER obesidad y nutrición, IRYCIS, Hospital Ramón y Cajal, Servicio Madrileño de Salud, Madrid, Spain; Johns Hopkins Bloomberg School of Public Health, United States of America

## Abstract

**Objective:**

To evaluate the incidence of diabetic retinopathy in patients with Type 2 Diabetes Mellitus, to identify the risk factors associated with the incidence of retinopathy and to develop a risk table to predict four-year retinopathy risk stratification for clinical use, from a four-year cohort study.

**Design:**

The MADIABETES Study is a prospective cohort study of 3,443 outpatients with Type 2 Diabetes Mellitus, sampled from 56 primary health care centers (131 general practitioners) in Madrid (Spain).

**Results:**

The cumulative incidence of retinopathy at four-year follow-up was 8.07% (95% CI = 7.04–9.22) and the incidence density was 2.03 (95% CI = 1.75–2.33) cases per 1000 patient-months or 2.43 (95% CI = 2.10–2.80) cases per 100 patient-years. The highest adjusted hazard ratios of associated risk factors for incidence of diabetic retinopathy were LDL-C >190 mg/dl (HR = 7.91; 95% CI = 3.39–18.47), duration of diabetes longer than 22 years (HR = 2.00; 95% CI = 1.18–3.39), HbA1c>8% (HR = 1.90; 95% CI = 1.30–2.77), and aspirin use (HR = 1.65; 95% CI = 1.22–2.24). Microalbuminuria (HR = 1.17; 95% CI = 0.75–1.82) and being female (HR = 1.12; 95% CI = 0.84–1.49) showed a non-significant increase of diabetic retinopathy. The greatest risk is observed in females who had diabetes for more than 22 years, with microalbuminuria, HbA1c>8%, hypertension, LDL-Cholesterol >190 mg/dl and aspirin use.

**Conclusions:**

After a four-year follow-up, the cumulative incidence of retinopathy was relatively low in comparison with other studies. Higher baseline HbA1c, aspirin use, higher LDL-Cholesterol levels, and longer duration of diabetes were the only statistically significant risk factors found for diabetic retinopathy incidence. This is the first study to demonstrate an association between aspirin use and diabetic retinopathy risk in a well-defined cohort of patients with Type 2 Diabetes Mellitus at low risk of cardiovascular events. However, further studies with patients at high cardiovascular and metabolic risk are needed to clarify this issue.

## Introduction

Diabetic Retinopathy (DR) is the most common microvascular complication in diabetes mellitus (DM). Worldwide, it remains a significant cause of acquired visual loss and blindness in the 20 to 60 years-old age group [1]–[3].

In an effort to detect DR at an optimal stage for intervention, the American Diabetes Association (ADA) recommends that, after the diagnosis of Type 2 DM (T2DM), patients should receive an initial dilated and comprehensive eye examination by an ophthalmologist, and subsequent annual examinations. Less frequent exams, every 2 or 3 years, may be considered following one or more normal eye exams [4]. However, initial data from the Health Plan Employer Data and Information Set (HEDIS) indicate that, in reality, only 35% to 50% of patients between 30 and 64 years-old receive the annual eye examination [5].

Presence of DR is not only linked to an increased risk of vision loss, but also a two to three-fold excess risk of coronary disease [6]–[9] and ischemic stroke [10].

Identifying individuals at risk of DR in order to establish preventive and therapeutic strategies is a highly important public health issue. Risk scores for DR, based on simple anthropometric and demographic variables, have been set to identify type 1 diabetes patients with high risk of DR [11]. Recently, cross-sectional studies with the same aim have also been published [12], but to our knowledge, no studies have been carried out in patients with T2DM.

We conducted a cohort study with a Spanish population with T2DM at 56 primary health care centers in Madrid, in order to evaluate the incidence of DR over four years, to identify the risk factors associated with incidence of DR, and to develop a risk table to predict four-year DR risk stratification for clinical use.

## Materials and Methods

### Study Population and Design

The Madrid Diabetes Study (MADIABETES Study) is a prospective cohort study of 3,443 T2DM outpatients, sampled from 56 primary health care centers in the metropolitan area of Madrid (Spain). Study participants were selected by simple random sampling by participating general practitioners (n = 131), using the list of patients with a T2DM diagnosis in their computerized clinical records. Using diabetes diagnosis in computerized clinical records for epidemiological studies has been validated in our setting [13].

**Figure 1:**
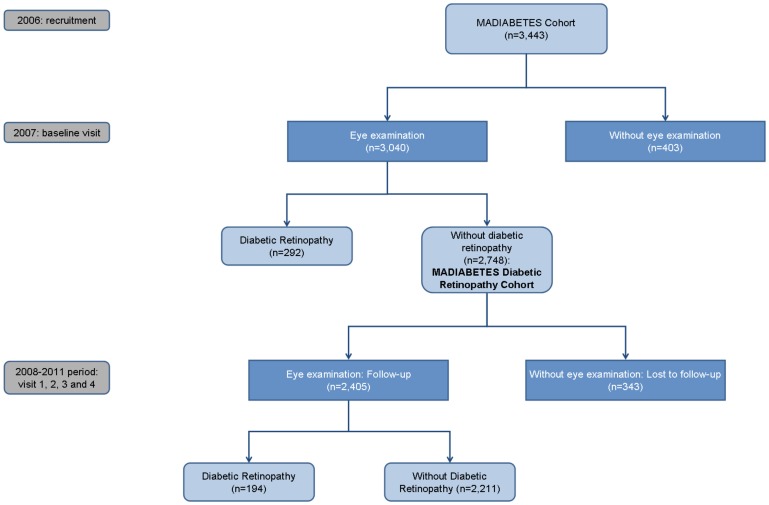
Flow diagram of the MADIABETES cohort.

Data were collected by general practitioners at baseline visit (2007) and annually during the follow-up period (2008–2011). These data were recorded in electronic Case Report Forms. The flow diagram of participants is shown in Figure 1. Last observation carried forward (LOCF) was used to impute missing values for patients with incomplete data during follow-up period.

Inclusion criteria were: age >30 years-old, a previous diagnosis of T2DM and provision of written informed consent to take part in the study. Exclusion criteria were: type 1 DM and homebound patients. Patients without an eye examination at baseline visit were excluded from follow-up (n = 403), as were patients who had been previously diagnosed with DR (n = 292), leaving a final sample size of 2,748 patients. Three hundred and forty-three patients were excluded from the final analysis for not having a second eye examination during the follow-up period.

After collection, all data were internally audited to ensure quality. This involved the random selection of 50 participating general practitioners and the review of the clinical records they produced. There was a strong data consistency (higher than 88% for all variables).

We examined DR incidence (DR at visits 1 to 4 among individuals without DR at baseline visit). The median follow-up period for patients was 47.97 months (Interquartile range [IQR] = 11.99).

The study was approved by the Institutional Review Board of the Ramón y Cajal Hospital (Madrid), and conducted in accordance with the principles of the Declaration of Helsinki.

### Assessment of DR

Eye examinations were conducted by ten experienced ophthalmologists, through dilated pupils with a slit lamp biomicroscopy examination, with a 90-D handheld magnifier lens. DR was diagnosed by the presence of any of the following lesions: microaneurysm, intraretinal hemorrhage, venous beading, neovascularization, vitreous/preretinal hemorrhage, cotton wool spots, retinal thickening, and hard exudates. The stage of DR was based in the severity scale proposed by Wilkinson et al. [14]. This scale has the following categories: no retinopathy, nonproliferative DR (mild, moderate and severe), proliferative DR and Diabetic Macular Edema (retinal thickening and hard exudates in the posterior pole). This DR severity scale is based on the results of the ETDRS [15] and, therefore, relies on scientific evidence, pretending not to displace the original classification, but only to provide a basis for simple operation and adequate clinical practice [16]. In the absence of photographic records, it was difficult to determine the number of microaneurysms per quadrant, so we decided to simplify the category of nonproliferative DR in two gradations mild and moderate/severe.

### Clinical Examination and Biochemistry

All patients were subjected to anamnesis, a physical examination, and biochemical tests. The following variables were collected at baseline visit: age, gender and duration of DM (years). Further data was collected at baseline and each follow-up visit: fasting plasma glucose (FPG), glycated haemoglobin (HbA1c), systolic (SBP) and diastolic blood pressure (DBP), total cholesterol, triglycerides (TG), high-density lipoprotein cholesterol (HDL-C), low-density lipoprotein cholesterol (LDL-C), albuminuria, smoking status (current smoker, former smoker, non-smoker), use of hypoglycemic and cardiovascular medications (antihipertensyve agents, statins, aspirin), body mass index (BMI), and history of cardiovascular events (myocardial infarction or stroke) and hypertension.

BMI was calculated as weight/height2 (Kg/m2), and patients with a BMI ≥30 were considered obese. Blood pressure was measured using a checked, calibrated sphygmomanometer. History of hypertension was defined as BPS ≥140 mmHg or BPD ≥90 mmHg or via the use of antihypertensives. After a 5 minutes rest period, a first reading was taken, followed by a second reading 5 minutes later. The mean result was then calculated; a value of <140/90 mm Hg was taken to reflect good control of blood pressure. A baseline FPG level of <126 mg/dL and a HbA1c level of <7% were considered to represent good control of these variables, as were values of <150 mg/dL for TG, <200 mg/dL for total cholesterol, and <100 mg/dL for LDL-C. In females, HDL-C was considered under control when values >50 mg/dL were recorded; in males, values >40 mg/dL were considered satisfactory. Persistent microalbuminuria was defined as a urinary albumin excretion of 30–300 mg/24 in at least two of three consecutive samples. HbA1c was measured using high performance liquid chromatography (Diabetes Control and Complications Trial [DCCT]-aligned) [17].

Cardiovascular risk was calculated following the REGICOR formula (a calibration of the Framingham algorithm adapted for Spain) for each patient [18]. Patients with a cardiovascular risk of 10% over 10 years were considered moderate or high risk [19].

### Statistical Analysis

Descriptive data were expressed as mean and standard deviation, and median and IQR. Comparison of continuous variables between two groups was performed with Student’s t-test for data that were normally distributed, The Mann–Whitney U-test for non-normal distributions, and the chi-square test for categorical variables.

Incident DR was analyzed using adjusted hazard ratios (HR) and corresponding 95% Confidence Intervals (CI) were estimated using an extension of Cox proportional hazards models for time-dependant variables. The variables HbA1c, microalbuminuria, LDL-C, blood pressure and use of aspirin were included as time-dependant variables, while gender and duration of diabetes were included as fixed variables. Possible confounding factors, such as use of insulin or aspirin, were checked.

The predictive accuracy of multivariable Cox model was evaluated by the C-index, which is equivalent to the area under the receiver operating characteristic curve for binary dependent variables.

Finally, a probability table of DR risk [20] was obtained with the regression coefficients. A lower DR probability (5%) was calculated in the absence of risk variables and a higher one was calculated in the presence of all of them (98.3%). Between these extremes, a total of 576 combinations of DR probabilities were obtained.

The analysis of the results with hierarchical or multilevel models was unnecessary, as there is no evidence of the variance between the primary health care centers for the HbA1c variable being different from zero (p = 0.34); in addition, the Coefficient Correlation Intraclass has a value of 0.036.

All calculations were performed using SPSS v.19.0 software for Windows. Significance was set at p value <0.05 for differences with a probable type I error.

## Results

Table 1 shows the socio-demographic and clinical characteristics at baseline visit of the MADIABETES DR Cohort, including the follow-up patients (with second eye examination; n = 2,405) and the lost to follow-up patients (without second eye examination; n = 343). Mean age in this patient cohort was 67.8 years-old (SD = 10.6) and mean duration of DM was 7.7 years (SD = 7.1). The vast majority (91.3%) had a low risk (less or equal to 10 percent) of developing coronary events within 10 years.

**Table 1:** Baseline characteristics of MADIABETES Diabetic Retinopathy Cohort (N = 2,748), and mortality rate during follow-up.

	OverallN = 2,748	95% CI	Follow-upn = 2,405	Lost to follow-upn = 343	p value
**Sociodemographic variables**					
Female gender. (%)	49.6	47.8–81.5	49.4	51.6	0.436
Age (yr), mean (SD)	67.8 (10.6)	67.4–68.2	67.5 (10.6)	68.7 (11.5)	0.052
Duration of DM (yr), mean (SD)	7.7 (7.1)	7.4–8	7.6 (7.2)	7.6 (7)	0.861
Duration of DM (yr), median (IQR)	6 (7)	6–6	6.0 (7)	6.0 (7)	0.306
Current smoker. (%)	13.5	12.3–14.8	13.4	14.3	0.649
Former smoker. (%)	12.2	11–13.5	12.9	7.3	0.003
Non-Smoker. (%)	73.9	72.2–75.5	73.4	77.3	0.131
**Medication Profile**, (%)					
Oral antidiabetic	75	73.3–76.6	75.3	72.9	0.350
Insulin	17.4	16–18.9	17.1	19.3	0.329
Antihypertensive agents	84.2	82.7–85.5	84.6	81.0	0.093
Aspirin	50.7	48.8–52.6	50.8	49.7	0.708
Statins	72.2	70.4–73.9	73.2	65.1	0.002
**History of**. (%)					
Myocardial Infarction	8	7–9.1	7.9	8.7	0.589
Stroke	7,1	6,2–8.2	7.1	7.6	0.731
Hypertension	70	68.2–71.7	70.3	67.9	0.376
**Risk of developing coronary events**					
Adjusted REGICOR function 10-year risk, mean (SD)	5.8 (2.8)	5.7–5.9	5.8	5.8	0.929
Proportion patients with risk <5%	53.9	51.9–55.8	53.5	56.4	0.312
Proportion patients with risk 5–10%	37.4	35.5–39.3	37.8	34.4	0.220
Proportion patients with risk >10%	8.8	7.7–9.9	8.7	9.2	0.761
**Clinical Measurements**					
BMI (Kg/m^2^), mean (SD)	29.4 (5.4)	29.1–29.6	29.3 (5.3)	29.0 (5.4)	0.374
SBP (mmHg), mean (SD)	133.2 (13.3)	132.7–133.7	133.4 (13.3)	133.8 (14)	0.614
DBP (mmHg), mean (SD)	76.7 (7.9)	76.4–77.1	76.8 (8)	76.8 (7.9)	0.935
**Laboratory variables**					
FPG (mg/dl), mean (SD)	143.1(40.7)	141.5–144.7	142.8(40.3)	146.2(44.)	0.156
FPG (mg/dl), median (IQR)	136 (42)	134–137	135.0 (42)	139.0 (45)	0.217
Patients with HbA1c level <7, (%)	54.9	53.1–56.8	55.5	50.9	0.112
HbA1c (%), mean (SD)	7 (1.1)	6.9–7.1	7.0 (1.2)	7.0 (1.2)	0.844
HbA1c (%), median (IQR)	6.8 (1.3)	6.8–6.9	6.8 (1.3)	6.9 (1.5)	0.515
Total Cholesterol (mg/dl), mean (SD)	192 (35.6)	190.6–193.4	192.5(35.6)	188.9 (35)	0.088
LDL-C (mg/dl), mean (SD)	115.1(29.6)	113.9–116.2	115.3(29.7)	113.4(28.5)	0.269
HDL-C (mg/dl), mean (SD)	49 (12.6)	48.4–49.4	49.1 (12.7)	47.8(12.3)	0.087
Triglycerides (mg/dl), mean (SD)	144.4(83.5)	141.1–147.7	145.4(87)	145.1(93)	0.958
Triglycerides (mg/dl), median (IQR)	123 (78)	121–126	124 (78)	122 (77)	0.587
MAU, (%)	22.1	20.5–23.6	22.3	20.4	0.432
**All-Cause Mortality (%)**	4,9	41–5,7	3.8	12.5	<0.001

SD: Standard Deviation; IQR: Interquartile range; DM: Diabetes mellitus; BMI: Body mass index; SBP: Systolic Blood Pressure; DBP: Dyastolic Blood Pressure; FPG: Fasting plasma glucose; HbA1c: Glycated haemoglobin; LDL-C: low-density lipoprotein cholesterol; HDL-C: high-density lipoprotein cholesterol; MAU: Microalbuminuia.

Follow-up patients and lost to follow-up patients do not differ statistically in socio-demographic and clinical baseline variables except in age, proportion of former smokers and mortality rate during the 2008–2011 period. None of these factors was of a magnitude likely to affect the generality of the results.

For follow-up patients, data at baseline visit showed that individuals who developed DR (n = 194) had a higher duration of DM, higher HbA1c levels, and greater use of insulin and aspirin versus non incidence cases (n = 2,211) (Table 2).

**Table 2:** Baseline characteristics of follow-up patients (n = 2,405).

	OverallN = 2,405	95% CI	Diabetic Retinopathyn = 194	Without Diabetic Retinopathyn = 2,211	p value
**Sociodemographic variables**					
Female gender. (%)	49.4	47.4–51.4	51.5	49.2	0.524
Age (yr), mean (SD)	67.5 (10.6)	67.1–68.0	66.6 (11.2)	67.6 (10.5)	0.192
Duration of DM (yr), mean (SD)	7.6 (7.2)	7.3–7.9	9.4 (8.8)	7.4 (7.0)	0.003
Duration of DM (yr), median (IQR)	6.0 (7.0)	5.0–6.0	7.0 (10)	6.0 (7.0)	0.004
Current smoker. (%)	13.4	12.1–14.9	12.4	13.5	0.664
Former smoker. (%)	12.9	11.6–14.3	9.3	13.2	0.180
Non-Smoker. (%)	73.4	71.6–75.2	74.2	73.4	0.793
**Medication Profile**, (%)					
Oral antidiabetic	75.3	73.5–77.0	78.8	75	0.241
Insulin	17.1	15.6–18.7	29.5	16.0	<0.001
Antihypertensive agents	84.6	83.1–86.0	87.0	84.4	0.329
Aspirin	50.8	48.8–52.8	60.1	50.8	0.007
Statins	73.2	71.4–75.0	75.6	73	0.403
**History of**. (%)					
Myocardial Infarction	7.9	6.9–9.0	9.8	7.7	0.308
Stroke	7.1	6.1–8.2	7.2	7.1	0.933
Hypertension	70.3	68.4–72.1	72.7	70.1	0.444
					
**Risk of developing coronary events**					
Adjusted REGICOR function 10-year risk, mean (SD)	5.8 (2.8)	5.7–5.9	5.7	5.8	0.774
Proportion patients with risk <5%	53.5	51.4–55.6	54.6	53.4	0.747
Proportion patients with risk 5–10%	37.8	35.8–39.8	36.8	37.9	0.761
Proportion patients with risk >10%	8.7	7.6–10	8.6	8.7	0.962
**Anthropometric variables**					
BMI (Kg/m^2^), mean (SD)	29.3 (5.3)	29.1–29.5	29.4 (5.4)	29.3 (5.3)	0.825
SBP (mmHg), mean (SD)	133.4 (13.3)	132.8–133.9	134.0 (12.1)	133.3 (13.4)	0.509
DBP (mmHg), mean (SD)	76.8 (7.9)	76.5–77.1	76.1 (7.7)	76.9 (80.)	0.193
**Laboratory variables**					
FPG (mg/dl), mean (SD)	142.8 (40.3)	141.1–144.4	149.6 (55)	142.2 (38.8)	0.081
FPG (mg/dl), median (IQR)	135.0 (42.0)	133.0–136.0	134.5 (51)	135.0 (42)	0.465
Patients with HbA1c level <7, (%)	55.5	53.5–57.5	45.9	56.3	0.005
HbA1c (%), mean (SD)	7.0 (1.2)	6.9–7.1	7.4 (1.4)	7.0 (1.2)	<0.001
HbA1c (%), median (IQR)	6.8 (1.3)	6.8–6.9	7.1 (1.6)	6.8 (1.3)	<0.001
Total Cholesterol (mg/dl), mean (SD)	192.5 (35.6)	191.1–193.9	194.3 (39.3)	192.3 (35.2)	0.475
LDL-C (mg/dl), mean (SD)	115.3 (29.7)	114.1–116.5	117.9 (32.1)	115.1 (29.5)	0.220
HDL-C (mg/dl), mean (SD)	49.1 (12.7)	48.5–49.6	48.4 (12.2)	49.1 (12.8)	0.474
Triglycerides (mg/dl), mean (SD)	145.4 (86.9)	141.8–149.9	142.4 (83.1)	145.6 (87.3)	0.625
Triglycerides (mg/dl), median (IQR)	124.0 (78.0)	121.0–126.0	122.5 (71)	124 (79.3)	0.872
MAU. (%)	22.3	20.7–24.0	24.2	22.1	0.498

SD: Standard Deviation; IQR: Interquartile range; DM: Diabetes mellitus; BMI: Body mass index; SBP: Systolic Blood Pressure; DBP: Dyastolic Blood Pressure; FPG: Fasting plasma glucose; HbA1c: Glycated haemoglobin; LDL-C: low-density lipoprotein cholesterol; HDL-C: high-density lipoprotein cholesterol; MAU: Microalbuminuia.

The cumulative incidence of DR at four-years was 8.07% (95% CI = 7.04–9.22) and the incidence density was 2.03 (95% CI = 1.75–2.33) cases per 1,000 patient-months or 2.43 (95% CI = 2.10–2.80) cases per 100 patient-years.

Moreover, considering the stage of DR, 30 out of 194 cases (15.5%) were patients with diabetic macular edema; 96 (49.5%) and 68 (35.1%) had non-proliferative and proliferative DR, respectively. Of the 96 cases of non-proliferative DR, 85 cases were mild and 11 moderate or severe.

Additionally, no significant differences were seen in stage DR between patients treated with or without aspirin (p = 0,954). Also, there were significant differences in the duration of DM between strata formed by stage of DR. Thus, patients with macular edema (12.7 years, SD = 7.40) or non-proliferative DR (11.4 years, SD = 10.6) presented higher duration of DM than proliferative DR (6.9 years, SD = 6.9) (p<0.001). However, there were no significant differences between the HbA1c level and the stage of DR (p = 0.081).

The adjusted HR of associated risk factors for incidence of DR is shown in Table 3. The highest HR was LDL-C>190 mg/dl (HR = 7.91; 95% CI = 3.39–18.47). Furthermore, the other variables with the highest HR were duration of DM longer than 22 years (HR = 2.00; 95% CI = 1.18–3.39), HbA1c>8% (HR = 1.90; 95% CI = 1.30–2.77) and use of aspirin (HR = 1.65; 95% CI = 1.22–2.24). Being female (HR = 1.12; 95% CI = 0.84–1.49) and microalbuminuria (HR = 1.17; 95% CI = 0.75–1.82) were not significant. After adjustment for gender, duration of diabetes, hypertension and HbA1c levels, insulin was not significantly associated with DR.

**Table 3:** Associated Risk Factors for Incident Diabetic Retinopathy (n = 194) after four-year follow-up of 2,405 patients (Multivariable Cox Regression).

Variables	aHR	HR 95% CI	p value
**HbA1c**			
<7%	1		
7–8%	1.39	1.01–1.92	0.044
>8%	1.90	1.30–2.77	<0.001
**Microalbuminuria** (yes/no)	1.17	0.75–1.82	0.484
**Gender** (female/male)	1.12	0.84–1.49	0.451
**Hypertension** (yes/no)	0.95	0.70–1.29	0.745
**Duration of Diabetes Mellitus**			
0–6 years	1		
7–14 years	1.22	0.88–1.70	0.227
15–22 years	1.64	1.05–2.57	0.029
>22 years	2.00	1.18–3.39	0.010
**Aspirin** (yes/no)	1.65	1.22–2.24	<0.001
**LDL-Cholesterol**			
<100 mg/dl	1		
100–190 mg/dl	0.87	0.65–1.16	0.332
>190 mg/dl	7.91	3.39–18.47	<0.001

HbA1c: Glycated hemoglobin a1c; aHR: adjusted hazard ratios; CI: Confidence interval.

Finally, the development of a probability table of DR risk can be seen in Table 4. A lower DR probability is observed in men with DM for less than 7 years, without microalbuminuria, with HbA1c<7%, no hypertension, LDL-C between 100–190 mg/dl and who did not use aspirin regularly. The greatest risk is observed in females, with a duration of DM of more than 22 years, with microalbuminuria, HbA1c>8%, hypertension, LDL-C>190 mg/dl, and regular aspirin use.

**Table 4:** Risk table: probability of developing Diabetic Retinopathy in four-year (n = 2,405).

				Duration of Diabetes Mellitus (years)																							
Gender	Hypertension	LDL-Cholesterol	Use of Aspirin	0 to 6						7 to 14						15 to 22						> = 23					
				Microalbuminuria						Microalbuminuria						Microalbuminuria						Microalbuminuria					
				No			Yes			No			Yes			No			Yes			No			Yes		
				Glycated Hemoglobin			Glycated Hemoglobin			Glycated Hemoglobin			Glycated Hemoglobin			Glycated Hemoglobin			Glycated Hemoglobin			Glycated Hemoglobin			GlycatedHemoglobin		
				<7	7–8	>8	<7	7–8	>8	<7	7–8	>8	<7	7–8	>8	<7	7–8	>8	<7	7–8	>8	<7	7–8	>8	<7	7–8	>8
Male	Yes	<100	No	6.1	8.4	11.2	7.1	9.7	13.0	7.4	10.2	13.6	8.6	11.8	15.7	9.8	13.4	17.8	11.4	15.5	20.5	11.8	16.0	21.2	13.7	18.5	24.3
			Yes	9.8	13.4	17.8	11.4	15.5	20.5	11.9	16.2	21.4	13.8	18.7	24.5	15.7	21.1	27.6	18.1	24.3	31.5	18.7	25.1	32.5	21.5	28.7	36.9
		100–190	No	5.3	7.3	9.8	6.2	8.5	11.4	6.4	8.9	11.9	7.5	10.3	13.7	8.6	11.7	15.6	9.9	13.6	18.0	10.3	14.1	18.6	12.0	16.2	21.4
			Yes	8.6	11.8	15.6	10.0	13.6	18.1	10.4	14.2	18.8	12.1	16.4	21.6	13.7	18.6	24.4	15.9	21.4	27.9	16.4	22.1	28.8	18.9	25.4	32.9
		>190	No	39.2	49.9	61.0	44.1	55.5	66.8	45.6	57.1	68.4	50.9	62.9	74.1	55.8	68.0	78.8	61.6	73.6	83.7	63.0	74.9	84.8	68.7	80.2	89.0
			Yes	56.0	68.1	78.9	61.7	73.7	83.8	63.3	75.3	85.1	69.1	80.5	89.2	74.0	84.7	92.2	79.4	88.9	95.0	80.6	89.8	95.5	85.3	93.1	97.4
	No	<100	No	5.8	8.0	10.7	6.7	9.3	12.4	7.0	9.7	12.9	8.2	11.2	15.0	9.3	12.8	17.0	10.8	14.8	19.6	11.2	15.3	20.2	13.0	17.7	23.3
			Yes	9.4	12.8	17.0	10.9	14.8	19.6	11.4	15.5	20.4	13.2	17.8	23.5	14.9	20.2	26.4	17.3	23.2	30.2	17.9	24.0	31.2	20.6	27.5	35.4
		100190	No	**5.0**	6.9	9.3	5.9	8.1	10.8	6.1	8.4	11.3	7.1	9.8	13.1	8.1	11.2	14.9	9.5	12.9	17.2	9.8	13.4	17.8	11.4	15.5	20.5
			Yes	8.2	11.2	14.9	9.5	13.0	17.2	9.9	13.5	18.0	11.5	15.6	20.7	13.1	17.7	23.3	15.1	20.4	26.7	15.7	21.2	27.6	18.1	24.3	31.5
		>190	No	37.6	48.2	59.1	42.4	53.7	64.9	43.9	55.3	66.6	49.1	61.0	72.2	54.0	66.1	77.0	59.7	71.8	82.1	61.1	73.2	83.3	66.9	78.5	87.7
			Yes	54.1	66.2	77.2	59.8	71.9	82.2	61.5	73.5	83.6	67.2	78.9	87.9	72.2	83.2	91.2	77.7	87.6	94.2	79.0	88.6	94.8	83.9	92.1	96.8
Female	Yes	<100	No	6.8	9.3	12.4	7.9	10.8	14.4	8.2	11.3	15.0	9.6	13.1	17.3	10.9	14.8	19.6	12.6	17.1	22.6	13.1	17.7	23.3	15.1	20.4	26.7
			Yes	10.9	14.9	19.7	12.7	17.2	22.6	13.2	17.9	23.5	15.3	20.6	27.0	17.3	23.3	30.3	20.0	26.7	34.4	20.7	27.6	35.5	23.7	31.4	40.2
		100–190	No	5.9	8.1	10.9	6.9	9.4	12.6	7.2	9.8	13.1	8.3	11.4	15.2	9.5	13.0	17.2	11.0	15.0	19.9	11.4	15.6	20.6	13.2	18.0	23.6
			Yes	9.5	13.0	17.3	11.1	15.1	19.9	11.5	15.7	20.8	13.4	18.1	23.8	15.2	20.5	26.8	17.5	23.6	30.6	18.2	24.4	31.6	20.9	27.9	35.9
		>190	No	42.6	53.8	65.1	47.7	59.5	70.8	49.3	61.2	72.4	54.8	66.9	77.8	59.8	71.9	82.3	65.6	77.4	86.8	67.0	78.7	87.8	72.7	83.6	91.5
			Yes	60.0	72.1	82.4	65.7	77.5	86.9	67.4	79.0	88.1	73.1	83.9	91.7	77.8	87.7	94.2	82.8	91.4	96.5	84.0	92.2	96.9	88.3	94.9	**98.3**
	No	<100	No	6.4	8.9	11.9	7.5	10.3	13.7	7.8	10.7	14.3	9.1	12.4	16.5	10.4	14.2	18.8	12.0	16.4	21.6	12.5	16.9	22.3	14.4	19.5	25.6
			Yes	10.4	14.2	18.8	12.1	16.4	21.6	12.6	17.1	22.5	14.6	19.7	25.8	16.5	22.3	29.0	19.1	25.5	33.0	19.7	26.4	34.1	22.7	30.1	38.6
		100–190	No	5.6	7.7	10.4	6.5	9.0	12.0	6.8	9.4	12.5	7.9	10.9	14.5	9.1	12.4	16.5	10.5	14.3	19.0	10.9	14.9	19.7	12.6	17.1	22.6
			Yes	9.1	12.4	16.5	10.5	14.4	19.0	11.0	15.0	19.8	12.8	17.3	22.8	14.5	19.6	25.7	16.7	22.5	29.3	17.3	23.3	30.3	20.0	26.7	34.5
		>190	No	41.0	52.0	63.2	46.0	57.7	68.9	47.5	59.3	70.6	53.0	65.1	76.1	58.0	70.1	80.7	63.7	75.7	85.4	65.2	77.0	86.4	70.9	82.1	90.4
			Yes	58.1	70.2	80.8	63.9	75.8	85.5	65.5	77.3	86.7	71.2	82.4	90.6	76.1	86.4	93.4	81.3	90.3	95.8	82.4	91.1	96.3	86.9	94.1	97.9

Bold type indicated the minimum and maximum values.

The C-index for the DR model was 0.654 (95% CI = 0.609–0.699), reflecting moderate ability to discriminate between patients who did and did not have a DR outcome. However, the C-index must be interpreted with great caution, as it is not often constructed with time-dependent variables.

## Discussion

### Incidence of DR

In the MADIABETES Cohort, the incident rate (2.43 cases per 100 patients/year) and the cumulative incidence of DR (8.07%) were relatively low. This could answer to the routine clinical practice conditions of the study and not having a specially selected study population. The Rochester study [21], performed under routine conditions, shows a four-year cumulative incidence similar to that found in our study (6.1%). However, previous findings, as seen in the LALES study [22] and the San Luis Valley Diabetes Study [23], report higher rates of DR, between 3 and 3.5 times higher than our study. Both these studies had a higher proportion of patients with elevated glucose levels and who were being treated with insulin, what is important due to the relationship insulin and glucose levels have with DR [24].

In Spain, a T2DM cohort of 130 patients attending Alcañiz Hospital (Teruel, Spain) [25] and followed for a mean of 5.2 years, reported a DR cumulative incidence of 36.2%. However, patients had elevated baseline levels of HbA1c (HbA1c = 7.9) and the duration of DM was also high (9.2 years). The MADIABETES cohort had an optimal baseline glycemic control (mean HbA1c = 7%; 55.5% patients with HbA1c<7%), which is in contrast with other studies that report only 17% of patients having HbA1c levels under 7% [26]. Similarly, in a cross sectional study, Brown et al. [27] observed that patients with T2DM had lower prevalence rates of DR compared to patients in the WESDR cohort [28], which may reflect lower mean HbA1c levels (7.9% vs. 10.%). Also, in the Australian Diabetes Obesity and Lifestyle study [29], the baseline HbA1c was close to 6.5% and the 5-year cumulative incidence DR was 13.9%, which are similar results to our findings.

The estimated crude annual incidence of DR in population based studies ranges from 2.2/100 patient-years to 8.6/100 patient-years [22], [23], [25], [30]–[33]. This discordance among various studies could respond to differences in study populations, methods, and definitions. Studies in which DR was identified by grading of seven stereoscopic fundus photographs [28] reached higher rates of DR than those carried out with the use of 45 degree nonstereoscopic, nonmydriatic photographs [34]. Our study was carried out with the use of mydriatic ophthalmoscopy examination through dilated pupils with indirect ophthalmoscopy. The crude annual incidence of DR was 2.4/100 patient-years, which is very similar to results found in the Melbourne Visual Impairment study, performed with two 30 degrees stereoscopic fundus photographs [33].

Moreover, when comparing incidence rates in the various studies, it is important to keep in mind the known duration of DM at the time of inclusion. The mean baseline duration of DM in our patients was 7.7 years, which is lower than in other studies [25], [35]–[38].

The stage distribution of DR was different to that found in other studies conducted in Spain, where there was a lower prevalence of proliferative DR [39]–[40]. This might be due to differences in methodology (prevalence versus incidence) and different methods to detect DR. No significant differences were seen in HbA1c levels among the retinopathy groups as other studies have observed [41].

### Risk Factors

In our Cohort, the main variable associated with development of DR was LDL-C. Thus, high LDL-C levels (>190 mg/dl) have a significant association with DR incidence, which is similar to findings in earlier studies [12], [42], [43]. Additionally, LDL-C levels between 100 and 190 mg/dl have no significant protector effect over incidence of DR. However, other studies have demonstrated an increase of DR incidence with low LDL-C levels [44], [45].

Patients who developed DR have used aspirin in a higher percentage than patients who did not develop it (60.1% vs. 50.8%; p<0.001). A possible explanation could be the increased history of ischemic heart disease in those who developed DR. However, too many patients in both groups were taking aspirin, a factor that is not common in other T2DM cohorts [46], and there is not sufficient evidence of its use in primary prevention of cardiovascular events [46].

An unexpected finding has been aspirin as an independent predictor of DR. This could be due to an increased risk of retinal hemorrhage with aspirin use, but our data show that intraretinal hemorrhages were observed in non-proliferative diabetic retinopathy and vitreous/preretinal hemorrhage in proliferative diabetic retinopathy. Also, the patients with a higher use of aspirin had similar incidence of non-proliferative diabetic retinopathy than patients with a lower use of aspirin or without it.

Our findings are not consistent with randomized controlled clinical trials included in a systematic review [47], and we have not found cohort studies evaluating the effect of aspirin on the incidence of DR. Moreover, the long-term use of aspirin (10 years) has recently been associated with a small but statistically significant increase in the risk of incidence late and neovascular Age-related Macular Degeneration [48]. However, a recent meta-analysis has not confirmed these findings [49]. Nevertheless, aspirin is effective for the prevention of cardiovascular events in patients with a history of vascular disease and it is reasonable to consider aspirin as one of the potential therapies for cardiovascular disease risk reduction in patients with DM and elevated cardiovascular disease risk [50].

Our findings are consistent with other studies that have found the predictive value of HbA1c and duration of DM on the incidence of DR [12], [28], [30], [37], [38], [44], [51]–[61]. In this respect, the Barbados study demonstrated that, for each 1% increase in mean HbA1c level at baseline, the incidence of DR increased 30% (RR = 1.3; 95% CI = 1.2–1.5) [62]. In our study, results are even higher: when HbA1c increases from 7% to 8% DR incidence is 38%, when HbA1c increases from 7% to >8% DR incidence is 86%.

The relative contribution HbA1c levels have to the risk of DR in populations is known to range from 9% [63] to 11% [53]. Consequently, improvements in glycemic control reduce the substantial burden of DR in T2DM patients. Direct evidence to test this hypothesis is available from the U.K. Prospective Diabetes Study, which demonstrated that a 1% decrease in HbA1c equated to a 31% reduction in DR [64], and an intensive blood-glucose control by either sulphonylureas or insulin substantially decreased the risk of retinal photocoagulation by 37% [65]. Also, the Wisconsin Epidemiologic Study [36] showed that decreases in HbA1c over the first four-years of follow-up were associated with improvements in DR.

Numerous studies have shown that the longer duration of T2DM is statistically significantly associated with the incidence of DR [22], [29], [32], [36], [37], [58], [60], [66]–[69]. However, other studies have not found this relationship [23], [30], .

A causal relationship between DR and hypertension was strongly suggested from studies carried out in the 1980s [70] and from the results of the UKPDS 38 study [71]. However, the Appropriate Blood Pressure Control in Diabetes Trial (ABCD) [72] revealed no relationship between blood pressure and incidence of DR. Our results support this conclusion as other recent studies do [12], [35], [73].

Microalbuminuria has been described as an independent predictor of DR [43], [58], [70], [74]–[86]. However, our study did not note a significant increase of DR, only a slight trend towards DR (HR = 1.17, 95% CI = 0.75–1.82). Additionally, older age, raised blood pressure, and poor glycemic control, have been identified as predictors of microalbuminuria [81]. In our study, the prevalence of microalbuminuria is low compared with other published studies [87]–[90]. This low prevalence, good blood pressure and HbA1c control could be explained, in part, by the lack of association between microalbuminuria and DR.

### Risk Table

To our knowledge, this is the first study that has developed a simple risk table to predict four-year DR in patients with T2DM.

Taking a high-risk person as an example: female, hypertension, microalbuminuria positive, LDL-C>190 mg/dl, use of aspirin, duration of DM 9 years, and HbA1c>8%, the risk of developing DR at four-year follow-up is 91.7%. However, in the same patient, with HbA1c<7%, the risk is 73.1%. Thus, after changes in HbA1c from <7% to >8%, the increased risk is nearly 19%.

This risk table enables to calculate individual risk estimates and risk reduction strategies to identify, for example, controlling certain parameters such as HbA1c or LDL-C, or stopping treatment with aspirin. Therefore, it is particularly useful for general practitioners caring for patients with T2DM in our setting, as it allows the monitoring of risk over time.

### Strengths and Limitations

The strengths of our study include the use of a population based cohort, the prospective ascertainment of end points, and assessment of information on potentially confounding variables, which reduces the potential selection and confusion bias. We believe our results are applicable to other patients with T2DM. This is an essential issue considering the accuracy of predictive models tends to be lower when applied to other data different from the used to develop those models. We addressed this issue by penalizing model complexity and by choosing models that generalized best to cohorts omitted from the estimation procedure. Our database included patients from many primary health care centers in Madrid. The range of patients was broad: male and female, aged from 30 years to the elderly, and the major exposure categories were well represented. The severity of T2DM at baseline ranged from not measurable to very severe.

It is necessary to validate the multivariate model in a longer sample in order to be able to make accurate, reliable predictions, which is a limitation of the study. Also, concerning the assessment of DR, the distribution of patients in ten different clinics for ophthalmologic care, made no ??possible sharing the non-mydriatic retinal camera. Moreover, the ETRDRS scale classification was not commonly used by ophthalmologists working in Primary Health Care Setting.

## Conclusions

In summary, the cumulative incidence, after four-year follow-up, of developing DR lesions in our cohort of patients with T2DM was 8.07% (95% CI = 7.04–9.22) and the incidence density was 2.03 (95% CI = 1.75–2.33) cases per 1,000 patient-months or 2.43 (95% CI = 2.10–2.80) cases per 100 patient-years.

In this study, higher baseline HbA1c, aspirin use, higher LDL-C levels, and longer duration of diabetes were the only statistically significant risk factors found for DR incidence after four-year follow-up.

This is the first study to demonstrate an association between aspirin use and DR risk in a well-defined cohort of patients with T2DM at low risk of cardiovascular events. However, further studies with patients at high cardiovascular and metabolic risk are needed to clarify this issue.

## Supporting Information

Figure S1 **Receiver operating characteristic (ROC) curve in risk prediction of DR, using the Cox model.**
(TIFF)

Table S1 **Hazard ratio of Diabetic Retinopathy in each stratum variables identified in multivariable analysis (n = 2,405).**
(DOC)
