# Supplementary figures and images for: Four-Year Incidence of Diabetic Retinopathy in a Spanish Cohort: The MADIABETES Study
Source: PLoS One. 2013 Oct 17;8(10):e76417. doi: 10.1371/journal.pone.0076417 (PMC3798464; doi:10.1371/journal.pone.0076417)

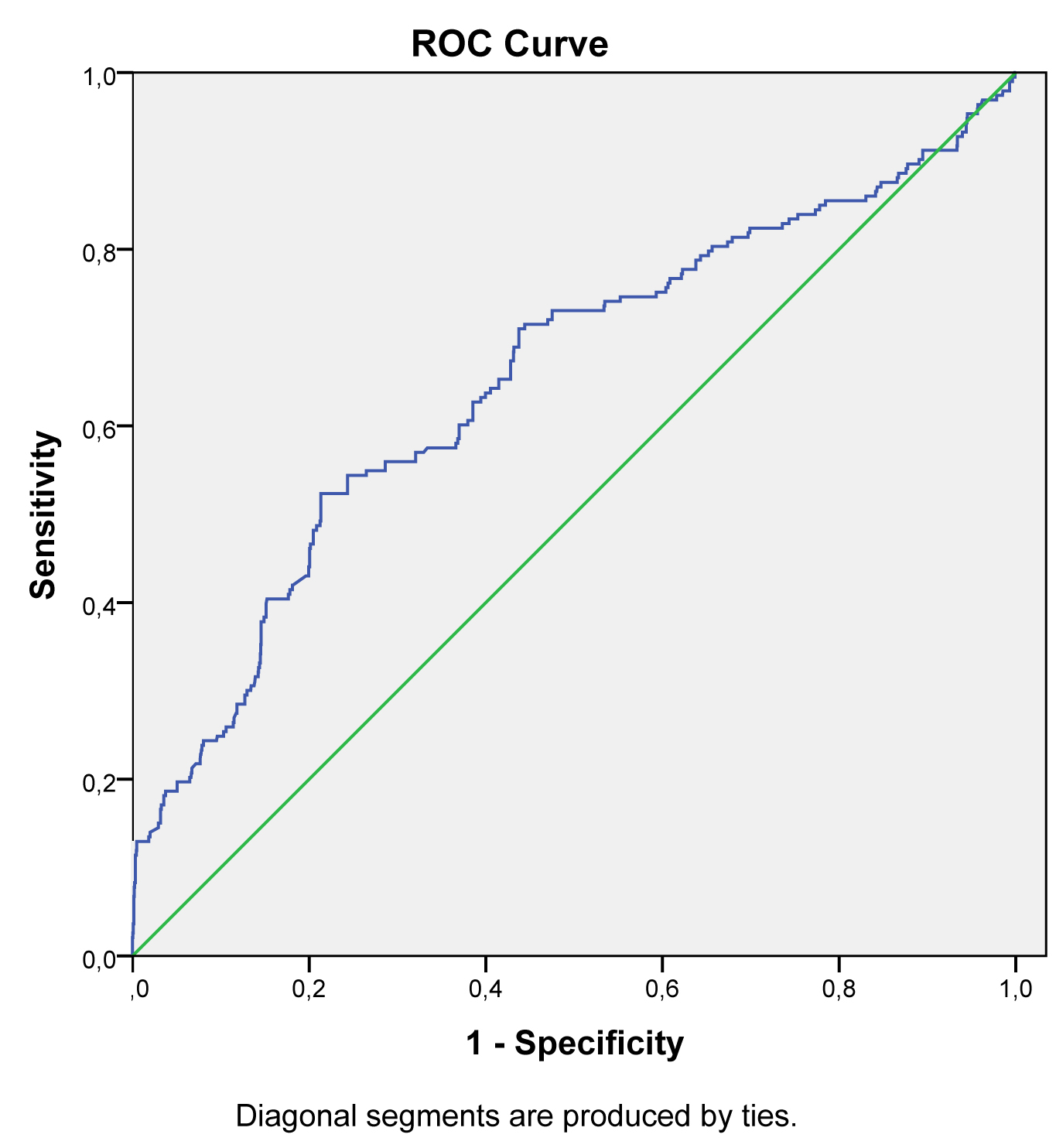

Supplement: Figure S1 — Receiver operating characteristic (ROC) curve in risk prediction of DR, using the Cox model. (TIFF) [file pone.0076417.s001.tiff]
