# Supplementary material for: Four-Year Incidence of Diabetic Retinopathy in a Spanish Cohort: The MADIABETES Study
Source: PLoS One. 2013 Oct 17;8(10):e76417. doi: 10.1371/journal.pone.0076417 (PMC3798464; doi:10.1371/journal.pone.0076417)
Supplement: Table S1 — Hazard ratio of Diabetic Retinopathy in each stratum variables identified in multivariable analysis (n = 2,405). (DOC) [file pone.0076417.s002.doc]

**Table S1. Hazard ratio of Diabetic Retinopathy in each stratum variables identified in multivariable analysis (n=2,405).**

|  |  |  |  | **Duration of Diabetes Mellitus** (years) | | | | | | | | | | | | | | | | | | | | | | | |
| --- | --- | --- | --- | --- | --- | --- | --- | --- | --- | --- | --- | --- | --- | --- | --- | --- | --- | --- | --- | --- | --- | --- | --- | --- | --- | --- | --- |
| Gender | Hypertension | LDL-Cholesterol | Use of Aspirin | 0 to 6 | | | | | | 7 to 14 | | | | | | 15 to 22 | | | | | | >=23 | | | | | |
|  |  |  |  | **Microalbuminuria** | | | | | | **Microalbuminuria** | | | | | | **Microalbuminuria** | | | | | | **Microalbuminuria** | | | | | |
|  |  |  |  | No | | | Yes | | | No | | | Yes | | | No | | | Yes | | | No | | | Yes | | |
|  |  |  |  | **Glycated**  **Hemoglobin** | | | **Glycated Hemoglobin** | | | **Glycated Hemoglobin** | | | **Glycated**  **Hemoglobin** | | | **Glycated**  **Hemoglobin** | | | **Glycated**  **Hemoglobin** | | | **Glycated**  **Hemoglobin** | | | **Glycated**  **Hemoglobin** | | |
|  |  |  |  | <7 | 7- 8 | >8 | <7 | 7- 8 | >8 | <7 | 7- 8 | >8 | <7 | 7- 8 | >8 | <7 | 7- 8 | >8 | <7 | 7- 8 | >8 | <7 | 7-8 | >8 | <7 | 7-8 | >8 |
| Male | Yes | < 100 | No | 1.2 | 1.7 | 2.3 | 1.4 | 2.0 | 2.7 | 1.5 | 2.1 | 2.8 | 1.7 | 2.4 | 3.3 | 2.0 | 2.8 | 3.8 | 2.3 | 3.2 | 4.4 | 2.4 | 3.4 | 4.6 | 2.8 | 3.9 | 5.4 |
|  |  |  | Yes | 2.0 | 2.8 | 3.8 | 2.3 | 3.3 | 4.4 | 2.4 | 3.4 | 4.6 | 2.9 | 4.0 | 5.4 | 3.3 | 4.6 | 6.2 | 3.8 | 5.4 | 7.3 | 4.0 | 5.6 | 7.6 | 4.7 | 6.5 | 8.9 |
|  |  | 100-190 | No | 1.0 | 1.5 | 2.0 | 1.2 | 1.7 | 2.3 | 1.3 | 1.8 | 2.4 | 1.5 | 2.1 | 2.8 | 1.7 | 2.4 | 3.3 | 2.0 | 2.8 | 3.8 | 2.1 | 2.9 | 4.0 | 2.5 | 3.4 | 4.6 |
|  |  |  | Yes | 1.7 | 2.4 | 3.3 | 2.0 | 2.8 | 3.8 | 2.1 | 2.9 | 4.0 | 2.5 | 3.5 | 4.7 | 2.8 | 4.0 | 5.4 | 3.3 | 4.6 | 6.3 | 3.5 | 4.8 | 6.6 | 4.0 | 5.6 | 7.7 |
|  |  | > 190 | No | 9.6 | 13.3 | 18.2 | 11.2 | 15.6 | 21.2 | 11.7 | 16.3 | 22.2 | 13.7 | 19.1 | 26.0 | 15.7 | 21.9 | 29.8 | 18.4 | 25.7 | 34.9 | 19.1 | 26.7 | 36.3 | 22.4 | 31.2 | 42.5 |
|  |  |  | Yes | 15.8 | 22.0 | 30.0 | 18.5 | 25.8 | 35.1 | 19.3 | 26.9 | 36.7 | 22.6 | 31.5 | 42.9 | 26.0 | 36.2 | 49.3 | 30.4 | 42.4 | 57.6 | 31.6 | 44.0 | 59.9 | 37.0 | 51.5 | 70.1 |
|  | No | > 190 | No | 1.1 | 1.6 | 2.2 | 1.3 | 1.9 | 2.5 | 1.4 | 2.0 | 2.7 | 1.6 | 2.3 | 3.1 | 1.9 | 2.6 | 3.6 | 2.2 | 3.1 | 4.2 | 2.3 | 3.2 | 4.4 | 2.7 | 3.7 | 5.1 |
|  |  |  | Yes | 1.9 | 2.6 | 3.6 | 2.2 | 3.1 | 4.2 | 2.3 | 3.2 | 4.4 | 2.7 | 3.8 | 5.2 | 3.1 | 4.3 | 5.9 | 3.7 | 5.1 | 6.9 | 3.8 | 5.3 | 7.2 | 4.4 | 6.2 | 8.4 |
|  |  | 100-190 | No | **1.0** | 1.4 | 1.9 | 1.2 | 1.6 | 2.2 | 1.2 | 1.7 | 2.3 | 1.4 | 2.0 | 2.7 | 1.6 | 2.3 | 3.1 | 1.9 | 2.7 | 3.6 | 2.0 | 2.8 | 3.8 | 2.3 | 3.2 | 4.4 |
|  |  |  | Yes | 1.6 | 2.3 | 3.1 | 1.9 | 2.7 | 3.6 | 2.0 | 2.8 | 3.8 | 2.4 | 3.3 | 4.5 | 2.7 | 3.8 | 5.1 | 3.2 | 4.4 | 6.0 | 3.3 | 4.6 | 6.2 | 3.8 | 5.4 | 7.3 |
|  |  | > 190 | No | 9.1 | 12.7 | 17.2 | 10.6 | 14.8 | 20.2 | 11.1 | 15.5 | 21.1 | 13.0 | 18.1 | 24.7 | 15.0 | 20.8 | 28.4 | 17.5 | 24.4 | 33.2 | 18.2 | 25.3 | 34.5 | 21.3 | 29.7 | 40.4 |
|  |  |  | Yes | 15.0 | 20.9 | 28.5 | 17.6 | 24.5 | 33.3 | 18.4 | 25.6 | 34.8 | 21.5 | 30.0 | 40.8 | 24.7 | 34.4 | 46.8 | 28.9 | 40.2 | 54.8 | 30.0 | 41.8 | 56.9 | 35.1 | 49.0 | 66.6 |
| Female | Yes | < 100 | No | 1.4 | 1.9 | 2.6 | 1.6 | 2.2 | 3.0 | 1.7 | 2.3 | 3.1 | 1.9 | 2.7 | 3.7 | 2.2 | 3.1 | 4.2 | 2.6 | 3.6 | 4.9 | 2.7 | 3.8 | 5.1 | 3.2 | 4.4 | 6.0 |
|  |  |  | Yes | 2.2 | 3.1 | 4.2 | 2.6 | 3.6 | 4.9 | 2.7 | 3.8 | 5.2 | 3.2 | 4.4 | 6.1 | 3.7 | 5.1 | 7.0 | 4.3 | 6.0 | 8.1 | 4.5 | 6.2 | 8.5 | 5.2 | 7.3 | 9.9 |
|  |  | 100-190 | No | 1.2 | 1.6 | 2.2 | 1.4 | 1.9 | 2.6 | 1.4 | 2.0 | 2.7 | 1.7 | 2.3 | 3.2 | 1.9 | 2.7 | 3.6 | 2.3 | 3.1 | 4.3 | 2.3 | 3.3 | 4.4 | 2.7 | 3.8 | 5.2 |
|  |  |  | Yes | 1.9 | 2.7 | 3.7 | 2.3 | 3.1 | 4.3 | 2.4 | 3.3 | 4.5 | 2.8 | 3.9 | 5.2 | 3.2 | 4.4 | 6.0 | 3.7 | 5.2 | 7.0 | 3.9 | 5.4 | 7.3 | 4.5 | 6.3 | 8.6 |
|  |  | > 190 | No | 10.7 | 14.9 | 20.3 | 12.5 | 17.4 | 23.7 | 13.1 | 18.2 | 24.8 | 15.3 | 21.3 | 29.0 | 17.6 | 24.5 | 33.3 | 20.6 | 28.7 | 39.0 | 21.4 | 29.8 | 40.5 | 25.0 | 34.9 | 47.4 |
|  |  |  | Yes | 17.6 | 24.6 | 33.5 | 20.6 | 28.8 | 39.1 | 21.6 | 30.1 | 40.9 | 25.3 | 35.2 | 47.9 | 29.0 | 40.4 | 55.0 | 34.0 | 47.3 | 64.4 | 35.3 | 49.2 | 66.9 | 41.3 | 57.5 | **78.3** |
|  | No | < 100 | No | 1.3 | 1.8 | 2.4 | 1.5 | 2.1 | 2.8 | 1.6 | 2.2 | 3.0 | 1.8 | 2.6 | 3.5 | 2.1 | 2.9 | 4.0 | 2.5 | 3.4 | 4.7 | 2.6 | 3.6 | 4.9 | 3.0 | 4.2 | 5.7 |
|  |  |  | Yes | 2.1 | 3.0 | 4.0 | 2.5 | 3.5 | 4.7 | 2.6 | 3.6 | 4.9 | 3.0 | 4.2 | 5.8 | 3.5 | 4.9 | 6.6 | 4.1 | 5.7 | 7.7 | 4.2 | 5.9 | 8.0 | 5.0 | 6.9 | 9.4 |
|  |  | 100-190 | No | 1.1 | 1.5 | 2.1 | 1.3 | 1.8 | 2.5 | 1.4 | 1.9 | 2.6 | 1.6 | 2.2 | 3.0 | 1.8 | 2.5 | 3.5 | 2.1 | 3.0 | 4.1 | 2.2 | 3.1 | 4.2 | 2.6 | 3.6 | 4.9 |
|  |  |  | Yes | 1.8 | 2.6 | 3.5 | 2.1 | 3.0 | 4.1 | 2.2 | 3.1 | 4.3 | 2.6 | 3.7 | 5.0 | 3.0 | 4.2 | 5.7 | 3.5 | 4.9 | 6.7 | 3.7 | 5.1 | 7.0 | 4.3 | 6.0 | 8.1 |
|  |  | > 190 | No | 10.2 | 14.2 | 19.3 | 11.9 | 16.6 | 22.5 | 12.4 | 17.3 | 23.6 | 14.5 | 20.3 | 27.6 | 16.7 | 23.3 | 31.7 | 19.5 | 27.2 | 37.1 | 20.3 | 28.3 | 38.5 | 23.8 | 33.1 | 45.1 |
|  |  |  | Yes | 16.8 | 23.4 | 31.8 | 19.6 | 27.3 | 37.2 | 20.5 | 28.6 | 38.9 | 24.0 | 33.5 | 45.5 | 27.6 | 38.4 | 52.3 | 32.3 | 44.9 | 61.2 | 33.5 | 46.7 | 63.6 | 39.2 | 54.7 | 74.4 |

Bold type indicated the minimum and maximum values.
